# Photobiomodulation and myofascial temporomandibular disorder: Systematic review and meta-analysis followed by cost-effectiveness analysis

**DOI:** 10.4317/jced.58084

**Published:** 2021-07-01

**Authors:** Ana-Paula-Taboada Sobral, Sergio-de Sousa Sobral, Thalita-Molinos Campos, Anna-Carolina-Ratto-Tempestini Horliana, Kristianne-Porta-Santos Fernandes, Sandra-Kalil Bussadori, Lara-Jansiski Motta

**Affiliations:** 1 Postgraduate Program in Biophotonics Applied to Health Sciences, UNINOVE - São Paulo, Brazil; 2 Postgraduate Program

## Abstract

**Background:**

Photobiomodulation (PBM) is a non-invasive and non-pharmacological treatment, which, has shown beneficial results in the treatment of temporomandibular disorders (TMD) related pain. This systematic review and meta-analysis study aimed to evaluate the efficacy of photobiomodulation in the treatment of myofascial pain associated with (TMD by analyzing randomized clinical trials published from 2007 to February 2019. The secondary objective of the study was to perform a cost-effectiveness analysis of TMD treatment with photobiomodulation in patients with myofascial pain.

**Material and Methods:**

International databases were used: Pubmed, Medline and Web of Science; the initial search raised 316 papers, and only 17 papers met the inclusion criteria for the systematic review (SR). Of these, only 04 papers met the inclusion criteria for meta-analysis: VAS data represented by numerical scores and placebo control group.

**Results:**

As for the wavelength, the most used value was 780nm (followed by 830nm. The most used treatment time was 4 offered treatments for 4 weeks; followed by 10 sessions. Regarding periodicity, 9 studies used 2 times a week. The meta-analysis showed that laser-treated groups had painful symptoms improvement that was superior to the control group (mean difference 1.49;95% CI = -1.67; -1.32). Laser therapy showed a cost-effectiveness of $1,464.28 by controlled pain intensity and placebo showed $2,866.20 by controlled pain intensity.

**Conclusions:**

The studies were considered to have moderate quality of evidence. Laser-treated groups had painful symptoms improvement that was superior to the control group and photobiomodulation was more cost-effective than placebo in patients with TMD and myofascial pain.

** Key words:**Temporomandibular disorder, Myofascial pain, Photobiomodulation, Placebo, Cost-effectiveness.

## Introduction

Temporomandibular disorder (TMD) is a term used to define clinical signs and symptoms affecting the masticatory muscles, the temporomandibular joint (TMJ) and associated structures (1-5). Among the most frequent signs and symptoms are masticatory muscle tenderness, pain in one or both TMJs, limited jaw movements, joint noise (5-7) and headache (5,8,9). TMD signs and symptoms are found at all ages; however, the prevalence of this disorder, considered low in children, increases with age in adolescents and young adults (10,11). Such disorders are a major cause of non-dental pain in the orofacial region, with 40% to 75% of nonpatient adult populations displaying at least one sign, and approximately 33% reporting at least one symptom of TMJ dysfunction (2). Among TMDs, the most common is myofascial pain, which causes pain and limited function, especially in chewing (4). Several resources, mainly for pain control, have been proposed for treatment such as occlusal splint, acupuncture, kinesiotherapy, massage therapy, postural training, psychotherapy, joint mobilizations, drug therapy, and laser therapy (12-13).

Low-level laser therapy (LLLT) or photobiomodulation (PBM) is a non-invasive and non-pharmacological treatment, which, according to several studies, has shown beneficial results in the treatment of TMD-related pain. (3-6,13-21).

The therapeutic effects of LLLT on TMD include inflammatory modulator and analgesic effects (4,5,7,21,22). Low-level lasers have demonstrated an ability to assist in the symptomatic treatment of pain, promoting a considerable degree of comfort to patients soon after its application. A major advantage of laser therapy for TMD is that it is a non-invasive, low-cost therapy and is currently widely used in dental practice, reducing the need for surgery or the use of drugs for pain relief and tissue regeneration. The use of laser therapy in patients with TMD has demonstrated pain relief minutes after application, promoting significant well-being. However, it is an adjunctive pain relief treatment due to the analgesic action of the laser which allows patients to resume their activities, providing them with greater convenience and better quality of life (4,5,21,22).

The main objective of this systematic review and meta-analysis was to assess the efficacy of photobiomodulation in the treatment of myofascial TMD by analyzing randomized clinical studies published within the period from 2007 to February 2019. The secondary objective was to conduct a cost-effectiveness (CE) study based on the results of the meta-analysis.

## Material and Methods

In order to maintain the methodological rigor of the systematic review and meta-analysis, the PRISMA (Preferred Reporting Items for Systematic Reviews and Meta-Analyzes) guide was used to aid the process, offering guidance to improve the quality of data reporting (23). The systematic review protocol was registered in PROSPERO CRD42019131016.

-Search Strategy

Search strategy was conducted with the assistance of an expert medical librarian. A systematic search of the literature was conducted using sources, PubMed, Web of Science and MEDLINE, between 2007 and February 2019. The search was restricted to papers written in English and limited to randomized clinical studies whose treated patients had a diagnosis of temporomandibular disorder with myofascial pain.

-Selection of studies 

**Figure 1:**
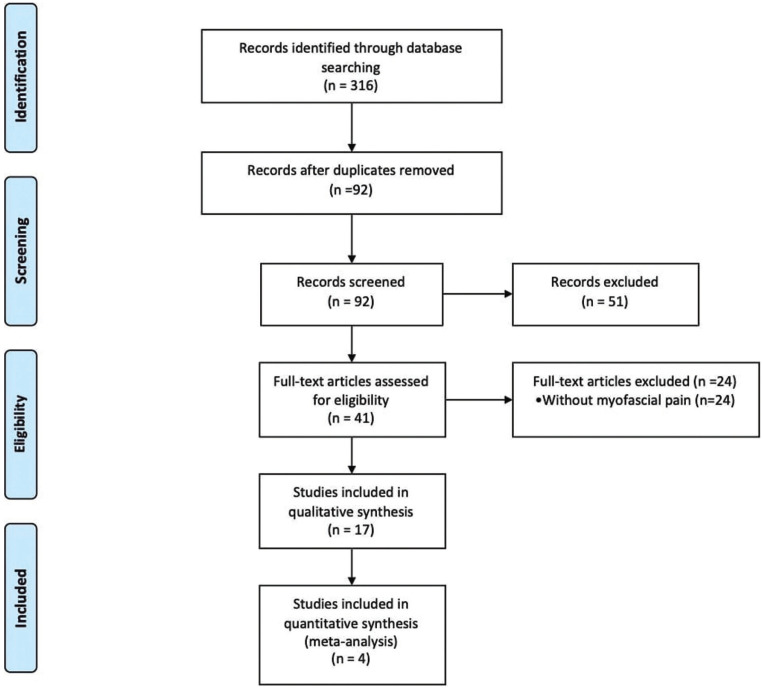
PRISMA flow chart.

An initial research using the keywords (“temporomandibular” OR “temporomandibular disorder” OR “temporomandibular joint” AND “temporomandibular joint” OR “low intensity laser therapy” OR “laser therapy” OR “photobiomodulation” OR “phototherapy” AND “myofascial pain”), in the databases (Pubmed, Medline and Web of Science) resulted in 316 studies. After reading the title and abstract, 17 papers that met the inclusion criteria were selected, as shown in Figure 1. The analysis was carried out by 2 trained reviewers and only randomized clinical studies were included, as they have higher level of evidence.

 -Inclusion Criteria

First stage, two reviewers independently screened all the titles and abstracts identified by the electronic searches to identify the potentially relevant articles to be retrieved. Second stage, full-text copies of these studies were assessed by the same two reviewers for inclusion using the eligibility criteria according to PICO strategy. The research question was established based on the structured PICO, in the systematic review is question format was as follows: “What is the effectiveness of photobiomodulation in the treatment of TMD in patients with myofascial pain when compared to placebo?”

P (population): Patient Diagnosis of temporomandibular disorder with myofascial pain

I (Intervention): Laser therapy

C (Comparison): Placebo

O (Outcomes): Pain (VAS)

In order to obtain homogeneity among the selected studies for the meta-analysis to be carried out, only the works that used the VAS scale (Visual Analogue Scale) were selected to evaluate the interference result. For the meta-analysis, only studies that used the simulated placebo in the control group and that showed numerically arranged data were included.

-Data Extraction

A data extraction form was designed to enable data extraction relating to the study author and year of publication, country where the study was conducted, number of subjects, type of laser, radiant exposure (J/cm2), wavelength (nm), power (mW), treatment duration and frequency. Data extraction was performed by 1 reviewer and checked for accuracy by a second reviewer.

-Assessment of Risk of Bias

The risk of bias was assessed using the revised Cochrane risk of bias tool (RoB 2.0) in accordance with the study design of the included trials. Risk of bias assessment of the included studies was undertaken by one reviewer and checked for agreement by a second reviewer.

-Quality of Evidence

The quality of evidence for the primary outcomes was assessed using GRADE criteria. Evidence was classified as either very low, low, moderate, or high quality determined by risk of bias, inconsistency of results across studies, indirectness of available evidence, imprecision of results, and publication bias.

-Cost-Effectiveness Analysis

Cost-effectiveness establishes whether a treatment should be implemented as a therapeutic measure, being calculated by the difference between the cost of two interventions proposed as treatment divided by the difference between its consequences (effectiveness) (24).

-Cost Analysis 

The cost of the visit (laser application session) was based on data presented by healthcare operators in Brazil and the treatment considered hospital costs in Brazil, following information from TUSS (Unified Terminology for Supplementary Health - Medical Procedures), with code and description: 31602215- LASER - PER SESSION (http://www.ans.gov.br/images/stories/Legislacao/in/anexo_in34_dides.pdf). An average of 2 sessions per week was considered, with 6 weeks of laser therapy, according to what was observed in the protocol by Sobral *et al*. (25).

In this research study, only direct medical costs were used, and the price of all materials used in the procedures was considered for calculation.

-Effectiveness Analysis

Treatment effectiveness in the systematic review followed by meta-analysis was measured by pain assessment using VAS data before and after treatment in the photobiomodulation and placebo groups.

-Data Analysis 

The meta-analysis of relative risk was carried out based on the selected dichotomous results. Heterogeneity between the studies was calculated using I2 statistics and the analysis used the fixed effects model in this study. The results were described with the respective 95% confidence interval (95% CI). Calculations were performed using the R software (The R Foundation for Statistical Computing, Austria). For all analyses, the level of significance was established as α= 0.05.

## Results

•Systematic Review 

-Characteristics of Included Studies

Papers were arranged in a (Table 1), mentioning authors, country of the authors, year of publication, the number of patients who entered the study, the type of laser device, energy density, and the time and periodicity of treatment.

**Table 1:**
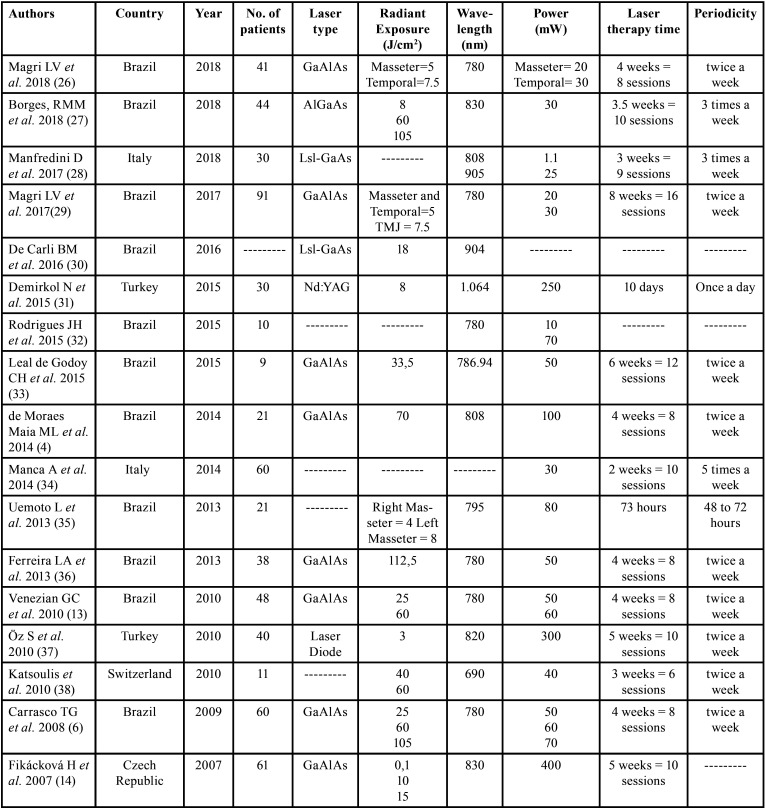
Papers included in Systematic Review.

As observed in the studies included in this work, there is no consensus regarding radiant exposure. Out of the 17 studies, 2 used: 5J/cm2 (masseter) and 7.5 J/cm2 (temporal) and 2 other studies, 25 J/cm2 and 60 J/cm2. As for the wavelength, the most used value was 780nm (35.29% - 6 studies), followed by 830nm (11.76% - 2 studies).

Based on the studies analyzed, the most used treatment time was 4 offered treatments for 4 weeks (4 studies); followed by 10 sessions, 3 studies. Regarding periodicity, 9 studies used 2 times a week.

-Systematic Review - Risk of Bias 

**Figure 2:**
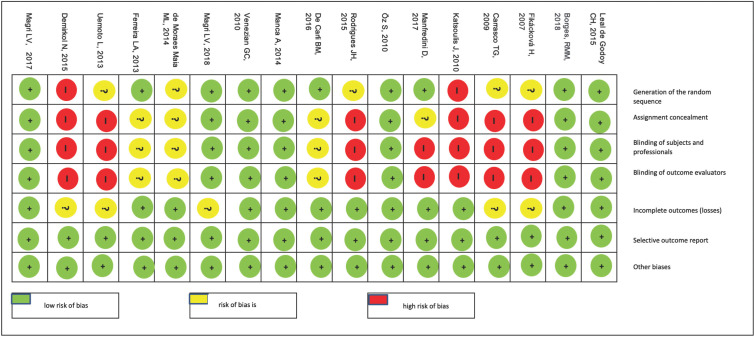
Risk of individual bias.

When assessing the risk of bias in the studies, Table 2 and Figure 2 demonstrate the risk of bias in each study individually, for each domain considered in the risk assessment, using RoB 2.0 tool of the Cochrane collaboration.

**Table 2:**
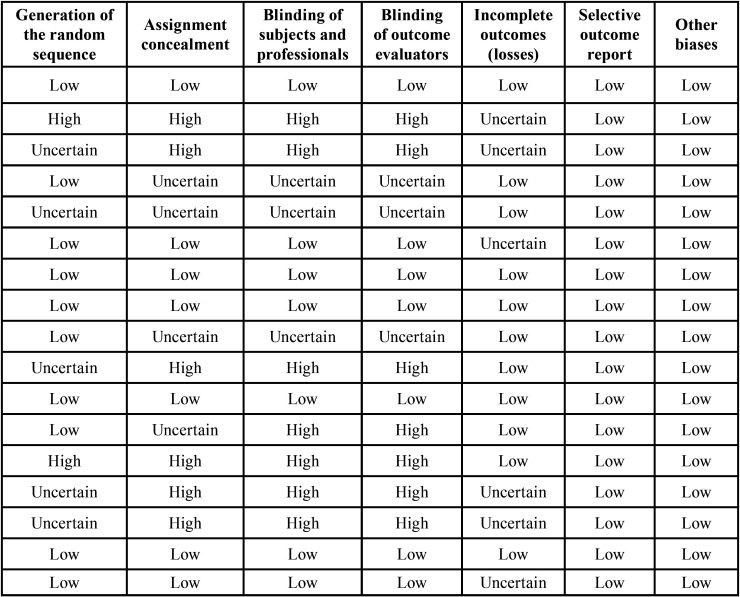
Risk of individual bias in the seventeen studies selected for the systematic review, for each domain of risk assessment of bias in randomized clinical trials using the Cochrane collaboration tool.

•Meta-Analysis 

The initial search resulted in 316 randomized clinical trials published from 2007 to February 2019, from which 17 papers were found and selected after evaluation by 2 reviewers, 4 of which met the inclusion criteria for meta-analysis: VAS data represented in numerical score and placebo control group.

-Quality of Evidence - GRADE 

The papers included in this study underwent assessment as to the quality of evidence. Thus, according to the GRADE (Grading of Recommendations Assessment, Developing and Evaluation), Table 3 shows the quality of evidence for each study included.

**Table 3:**
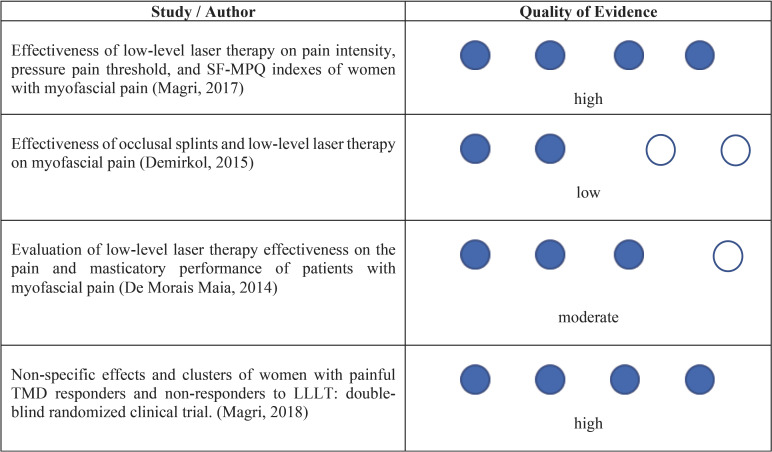
Evaluation of the Quality of Evidence from Studies.

It was observed that only 2 studies had a high degree of evidence quality, 1 had a moderate degree of evidence quality, and the other study had a low level of evidence quality. The studies were considered to have moderate and low quality of evidence because randomization and blinding were not well described and ensured in the methodology.

-Effect of Low-Level Laser Therapy on TMD

**Figure 3:**
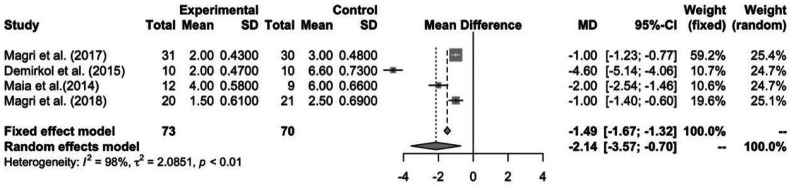
Forest Plot with Data from Meta-Analysis Studies.

In total, with the 4 studies included in this meta-analysis Magri *et al*. (26), Demirkol *et al*. (31), de Moraes Maia *et al*. (4) and Magri et. al (29), this study evaluated 143 patients. According to Figure 3, we can see that 73 patients were in the laser group, while 70 were in the control group (placebo). These patients were further divided in terms of event, and effectiveness was measured by means of the difference in the absolute mean of VAS scores before and after treatment (1.49).

For this analysis, pain was considered as the outcome measure, being assessed by the visual analogue scale. The 4 studies were grouped for meta-analysis. The forest plot (Fig. 3) describes the weighted meta-analysis regarding pain intensity in patients with TMD. Significant heterogeneity was observed among the studies (I2 = 98%; *p* <0.01) and a statistically significant difference was observed between the laser-treated group and the placebo group. The total mean difference was 1.49 (95% CI = -1.67; -1.32). The meta-analysis showed that laser-treated groups had painful symptoms improvement that was superior to the control group.

As for the mean difference analyzed separately for each study, the difference ranged from -1.00 to -4.60 on the pain scale. That is, in all studies it was possible to notice that the laser group shows a superior and statistically significant improvement in painful symptoms, which shows treatment effectiveness for cases of muscle pain in patients with TMD.

•Cost-Effectiveness 

For the cost-effectiveness analysis, the value of 1.49 was considered as effectiveness, which represents the difference in the absolute mean of VAS scores before and after treatment (1.49) and the costs were calculated for a total of 12 sessions.

Table 4 describes the costs per patient and also per group within the sample, with the total cost of 12 sessions.

**Table 4:**
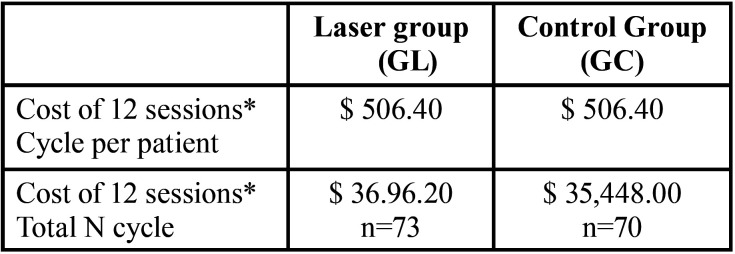
Description of treatment costs in the laser and control groups.

The incremental cost of the Pain outcome in this study is $992.75 per controlled pain intensity. The cost-effectiveness ratio for clinical treatment in the laser and placebo groups was $1,464.28 and $2,866.20 for controlled pain, respectively. The laser group being more cost-effective than the placebo group.

## Discussion

The analyzed papers in RS indicate that low-level laser has been increasingly used to treat patients with myofascial TMD due to its analgesic, regenerative and anti-inflammatory effects and also due to the conservative characteristic of treatment. The survey also showed that no agreement has yet been reached regarding the parameters used in the treatments and, therefore, we do not have a defined protocol for the treatment of myofascial TMD. This can make it difficult for the treatment to be standardized in public healthcare.

In addition, an aspect that made the analysis of the protocols used quite difficult is precisely because the authors did not provide all parameters for laser application. This occurred in 7 studies included in this study.

Of the 17 papers evaluated, we found a twice a week periodicity for laser therapy time in 10 studies, the minimum was 73 hours, and the maximum was 12 weeks. This study used the same periodicity and duration of treatment as the study by Leal de Godoy *et al*. (33).

The minimum parameter of 3 J/cm2 has managed to show satisfactory results in improving pain and also in opening the mouth of patients with myofascial TMD (37).

According to what was possible to observe in this meta-analysis, all the studies analyzed showed that the laser-treated group had statistically superior improvement in painful symptoms when compared to the placebo group.

Ahrari *et al*. (18) conducted a randomized, double-blind clinical trial with 20 female patients who had myogenic TMD. The patients were divided into two groups, the laser group and placebo. As a result, it was observed that there was a significant reduction in pain symptoms in the laser group and a significant increase in the mouth opening parameter (*p*<0.05). Statistically significant improvement was not seen in the placebo group. Thus, the authors concluded that LLLT can provide significant improvements in the level of pain and mouth opening in patients with myogenic TMD. The relative risk made by the proportion of all studies included in the meta-analysis was 1.49, using the fixed model, since the studies evaluated the same effect in different samples, through VAS. When assessing quality of evidence in the studies, only one study showed low quality, two showed high quality and one showed moderate quality of evidence.

If we analyze the relative risk of each study, the works by Magri *et al*. (26,29) were the ones with the lowest relative risk, 1.00 and their weights in the analysis were 59.2% and 19.6%, being the highest weights in the study. The work by Demirkol *et al*. (31) showed the highest relative risk, 4.60 and its relative weight in the analysis was 10.7% when compared to the placebo group. But, for the relative risk, analyzing all the studies.

When the quality of the evidence from the studies was considered, the studies by Magri *et al*. (26,29) showed high quality of evidence and that of Demirkol *et al*. (31) showed a low level of quality of evidence.

## Conclusions

According to what was observed in the studies analyzed through systematic review and meta-analysis, laser therapy is effective when compared to placebo and more cost-effective in the treatment of myofascial TMD.
